# Identification of Toxic Proteins Encoded by Mycobacteriophage TM4 Using a Next-Generation Sequencing-Based Method

**DOI:** 10.1128/spectrum.05015-22

**Published:** 2023-05-08

**Authors:** Chun-Liang Wang, Lan-Yue Zhang, Xin-Yuan Ding, Yi-Cheng Sun

**Affiliations:** a MOH Key Laboratory of Systems Biology of Pathogens, Institute of Pathogen Biology, and Center for Tuberculosis Research, Chinese Academy of Medical Sciences and Peking Union Medical College, Beijing, China; b Laboratory of Molecular Biology, Beijing Key Laboratory for Drug Resistance Tuberculosis Research, Beijing Chest Hospital, Capital Medical University, Beijing Tuberculosis and Thoracic Tumor Research Institute, Beijing, China; South China Sea Institute of Oceanology

**Keywords:** mycobacteriophage TM4, *Mycobacterium tuberculosis*, toxic genes, next-generation sequencing

## Abstract

Mycobacteriophages are viruses that specifically infect mycobacteria and which, due to their diversity, represent a large gene pool. Characterization of the function of these genes should provide useful insights into host-phage interactions. Here, we describe a next-generation sequencing (NGS)–based, high-throughput screening approach for the identification of mycobacteriophage-encoded proteins that are toxic to mycobacteria. A plasmid-derived library representing the mycobacteriophage TM4 genome was constructed and transformed into Mycobacterium smegmatis. NGS and growth assays showed that the expression of TM4 gp43, gp77, -78, and -79, or gp85 was toxic to M. smegmatis. Although the genes associated with bacterial toxicity were expressed during phage infection, they were not required for lytic replication of mycobacteriophage TM4. In conclusion, we describe here an NGS-based approach which required significantly less time and resources than traditional methods and allowed the identification of novel mycobacteriophage gene products that are toxic to mycobacteria.

**IMPORTANCE** The wide spread of drug-resistant Mycobacterium tuberculosis has brought an urgent need for new drug development. Mycobacteriophages are natural killers of M. tuberculosis, and their toxic gene products might provide potential anti-M. tuberculosis candidates. However, the enormous genetic diversity of mycobacteriophages poses challenges for the identification of these genes. Here, we used a simple and convenient screening method, based on next-generation sequencing, to identify mycobacteriophage genes encoding toxic products for mycobacteria. Using this approach, we screened and validated several toxic products encoded by mycobacteriophage TM4. In addition, we also found that the genes encoding these toxic products are nonessential for lytic replication of TM4. Our work describes a promising method for the identification of phage genes that encode proteins that are toxic to mycobacteria and which might facilitate the identification of novel antimicrobial molecules.

## INTRODUCTION

Tuberculosis (TB), caused by the bacillus Mycobacterium tuberculosis, caused 1.6 million deaths in 2021 (1). The emergence of multidrug-resistant (MDR) TB and extensively drug-resistant (XDR) TB and the slow development of new antibiotics provide significant challenges in the prevention and treatment of TB (1–3). Thus, new approaches for the development of antimycobacterial drugs are urgently needed. Mycobacteriophages are viruses that infect mycobacterial hosts. To date, more than 10,000 mycobacteriophages have been isolated using the prophage-free Mycobacterium smegmatis mc^2^155 host strain, and over 2,150 mycobacteriophage genomes have been completely sequenced (4). Apart from seven singleton strains that have no close relatives, mycobacteriophages are grouped into 31 clusters (5, 6). The enormous genetic diversity of mycobacteriophages is also reflected in the relatively large number of phage-encoded proteins and that most of their functions are unknown. Identification and characterization of Mycobacterium-killing or Mycobacterium-inhibiting gene products and their specific host targets could uncover new targets for the development of antimycobacterial drugs and the use of mycobacteriophages as therapeutic agents (7–12). In addition, small, toxic proteins could act as the templates for designing novel antimicrobial peptides (13–17).

A variety of mycobacteriophage gene products have been identified to be toxic to M. smegmatis (18, 19). This is usually determined by cloning and expressing individual mycobacteriophage genes in M. smegmatis and demonstrating the inhibition of host cell growth. Using this approach, 193 genes, encoded by 13 different mycobacteriophages, were evaluated, and 45 of the genes encoded toxic products (20). To simplify the procedure for identifying genes in mycobacteriophages encoding toxic proteins, Singh et al. (21) developed an approach that combined phage library construction and bioinformatic analysis. Using this method, four genes encoding toxic proteins were identified in the mycobacteriophages D29 and Che12 (21). However, this method failed to find any genes encoding toxic proteins in mycobacteriophages TM4, Che9c, and L5, suggesting limitations to the method.

Here, we describe a phage genomics approach, in combination with a next-generation sequencing (NGS)–based assay, to identify novel, toxic mycobacteriophage proteins. Using this method, we successfully identified gene products of mycobacteriophage TM4 with toxic effects against M. smegmatis and M. tuberculosis. We further verified the screening results and demonstrated that these toxic genes are not necessary for the lytic growth of phage TM4. This methodology could be used to identify genes encoding toxic proteins in other phages and thus could enhance our understanding of host-phage interactions and facilitate the development of new antimicrobial molecules.

## RESULTS

### Screening of mycobacteriophage TM4 for bacteriostatic gene products using an NGS-based assay.

**FIG 1:**
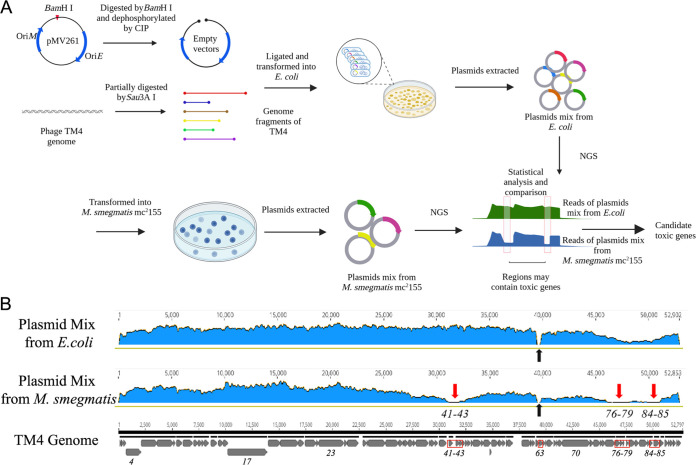
Screening for toxic mycobacteriophage TM4 proteins. (A) Overview of the NGS-based screening method. The TM4 phasmid genome was digested with Sau3AI and ligated into the shuttle expression plasmid pMV261.Using NGS, candidate genes associated with bacterial toxicity were initially screened by comparing the reads of TM4 genes in plasmids libraries extracted from M. smegmatis and E. coli. The image was created with BioRender. (B) Read peaks of each gene in recombinant plasmid mixtures from E. coli (upper panel) and M. smegmatis (middle panel) aligned with the TM4 genome (lower panel). Red arrows indicate regions exhibiting significant differences between the two plasmid mixtures. The black arrow indicates the region absent in the phasmid phAE87, compared with the original phage TM4.

TM4 is a subcluster K2 mycobacteriophage which can effectively infect M. tuberculosis (22). TM4 has 92 predicted genes, most of which have unknown functions (23, 24). To identify genes that encode proteins toxic to mycobacteria, a genomic library of TM4 was generated by insertion of 3 to 10 kb from TM4 phagemid phAE87 DNA into the shuttle plasmid pMV261 (25) (Fig. 1A). The ligated plasmid mixtures were transformed into Escherichia coli TransT1, and approximately 10^4^ transformants were obtained. Recombinant plasmids were extracted from E. coli and further transformed into M. smegmatis mc^2^155, resulting in approximately 10^4^ transformants. The plasmid mixtures from E. coli and M. smegmatis were compared using NGS analysis (Fig. 1A). When the plasmid mixtures from E. coli were compared with those from M. smegmatis, a significant reduction in reads for three regions of phage TM4 was observed in the M. smegmatis-plasmid mixtures (Fig. 1B; see also Table S1 in the supplemental material). The first region contained genes *41* to *43*, in which gene *43* encodes the Xis protein, which was reported to be toxic gene in mycobacteriophage D29 (21, 26). The other regions contained genes *76* to *79*, *84*, and to *85*, whose functions are currently unknown. Interestingly, the region with genes *77* to *85* also had relatively low reads in the plasmid mixtures from E. coli, indicating that this region may also be toxic to E. coli.

### Identification of TM4 bacteriostatic genes.

**FIG 2:**
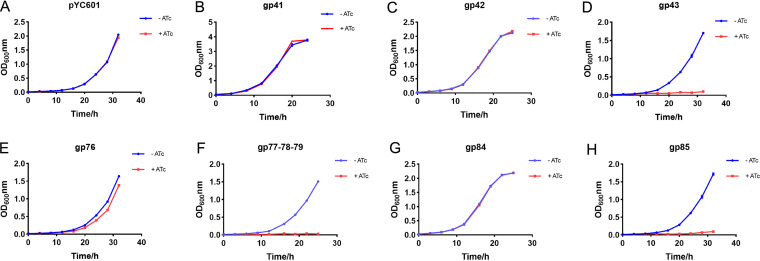
Growth curves of M. smegmatis carrying an empty pYC601 plasmid (A) or pYC601 containing the cloned mycobacteriophage genes specified (B to H). Blue and red lines indicate OD_600_ values in the absence or presence of ATc at a concentration of 100 ng/mL, respectively. The experiments were repeated three times with similar results, and the growth curve from one experiment is shown.

To identify the TM4 genes associated with mycobacterial toxicity, the genes identified in the screening assay were cloned into plasmid pYC601, an E. coli-Mycobacterium shuttle vector with an inducible promoter, and expressed in M. smegmatis. Induced expression of genes *41*, *42*, and *84* did not have any antibacterial or bactericidal effects on M. smegmatis (Fig. 2B, C, and G), whereas expression of gene *43* or *85* significantly inhibited the growth of M. smegmatis (Fig. 2D and H).The toxicity of genes *43* and *85* might account for the low level of reads of genes *41*, *42*, and *84* observed in the M. smegmatis plasmid mixtures. Expression of gene *77*, *78*, or *79* alone did not significantly affect the growth of M. smegmatis (data not shown). However, coexpression of genes *77*, *78*, and *79* significantly inhibited the growth of M. smegmatis (Fig. 2F). In addition, expression of gene *76* alone slightly repressed the growth of M. smegmatis (Fig. 2E). Next, we evaluated the toxicity of these genes in M. tuberculosis. Only a few transformants could be obtained when the plasmid for expression of genes *77*, *78*, and *79* was transformed to M. tuberculosis, indicating coexpression of genes *77*, *78*, and *79* might be strongly toxic to M. tuberculosis (Fig. S1A). Expression of gene *85* showed moderate repression for the growth of M. tuberculosis. However, expression of genes *43* and *76* did not significantly affect the growth of M. tuberculosis (Fig. S1B), indicating that gp43 and gp76 might not be toxic to M. tuberculosis. To further evaluate the phenotypic effects of these toxic genes on Mycobacterium, we examined the morphology of M. smegmatis under a microscope. Consistent with a previous report (21), expression of gene *43* in M. smegmatis caused long and filamentous cells (Fig. S2). However, expression of other toxic genes did not have a significant effect on the bacterial morphology (Fig. S2). Taken together, these results confirmed the screening results and suggest our NGS-based toxic gene screening method is effective in identifying genes whose products are antimycobacterial.

**FIG 3:**
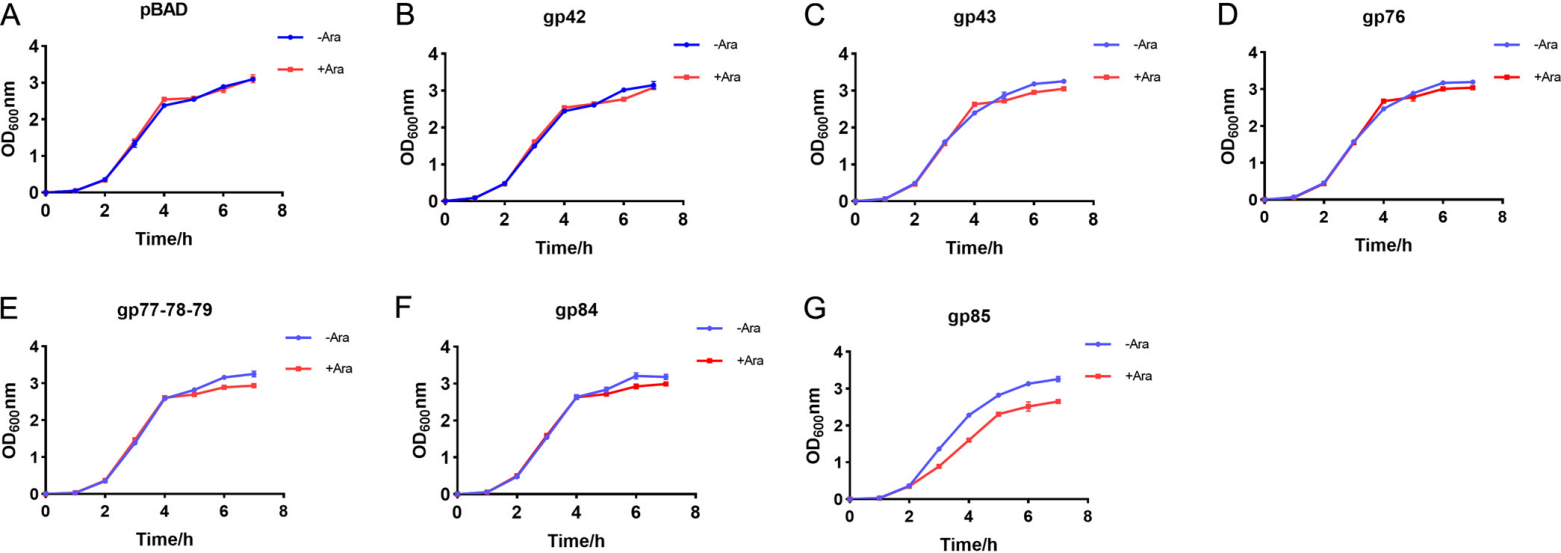
Growth curves of E. coli carrying an empty pBAD vector (A) or pBAD containing the cloned mycobacteriophage genes specified (B to G). Blue and red lines indicate OD_600_ values in the absence or presence of the inducer arabinose, at a concentration of 0.1%, respectively. The experiments were repeated three times with similar results, and the growth curve from one experiment is shown.

To further test whether these inhibitory effects were specific to mycobacteria, the same genes were expressed in E. coli. Expression of genes *43*, *76*, or *77*, *78*, and *79* did not affect E. coli growth, whereas expression of gene *85* had a slight affect (Fig. 3A to G). Consistent with this, spot plate experiments showed that expression of gene *85*, but not that of the other genes, in E. coli reduced the colony count (Fig. S3A and B). The toxicity associated with gene *85* in E. coli might account for the low level of reads observed for the region with genes *77* to *85* in the E. coli-plasmid mixtures (Fig. 1B).

### The genes associated with bacteriostatic effects are not required for lytic growth of mycobacteriophage TM4.

**FIG 4:**
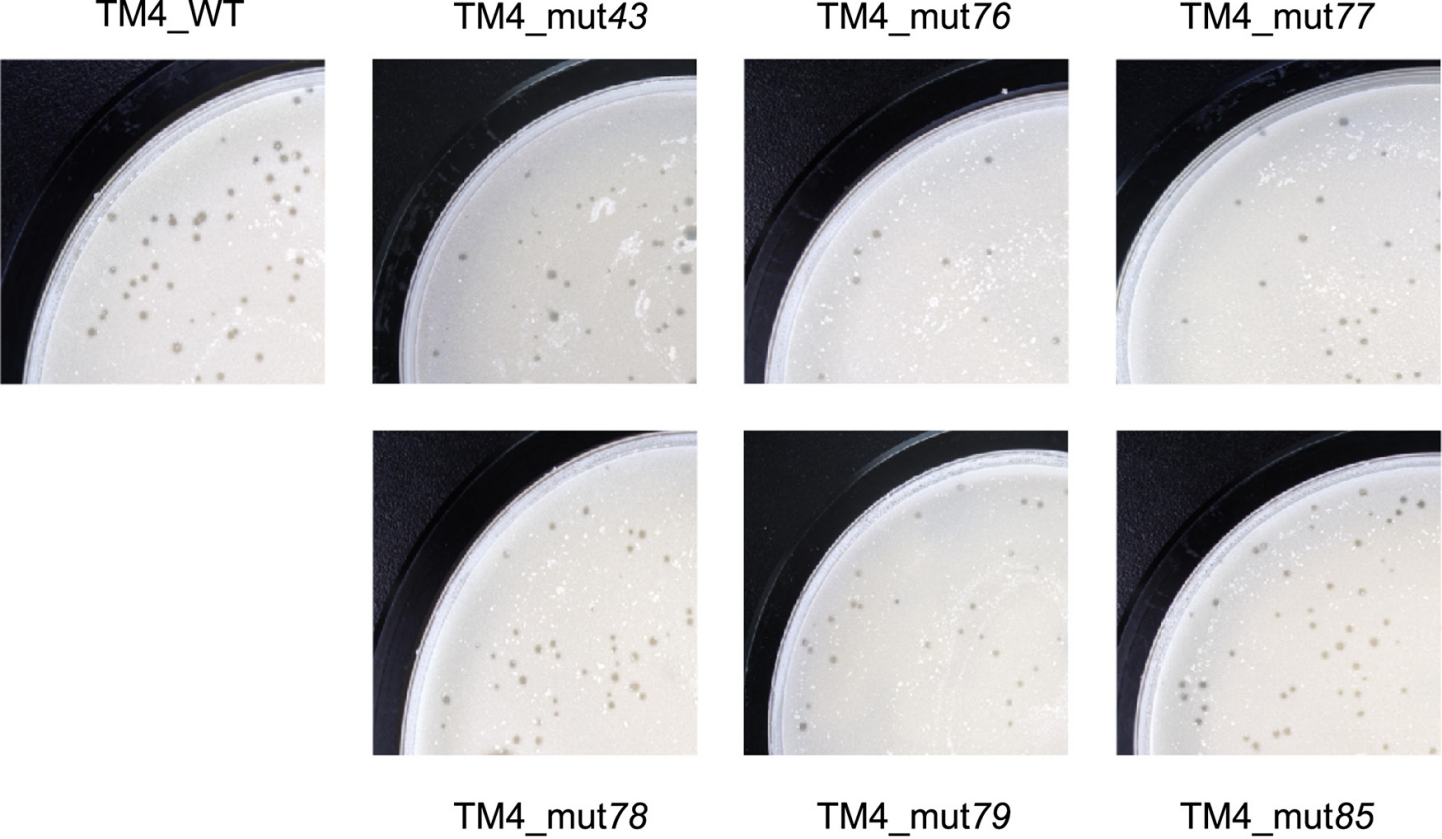
Mycobacteriophage genes associated with mycobacterial growth inhibition are not required for lytic growth of mycobacteriophage TM4. Wild-type TM4 phasmid and derivatives containing mutations in the genes specified were electroporated into M. smegmatis mc^2^155, and phage plaque formation was observed using a bilayer agar method.

To determine whether the genes associated with bacteriostatic effects in mycobacteria are required for lytic growth of phage TM4, mutations were separately introduced into each of these genes in the TM4 phasmid phAE87 (25). The mutated phasmids were electroporated into M. smegmatis and assessed for their ability to induce lytic growth. All the phage mutants formed plaques of similar size and number to those of wild-type TM4 (Fig. 4). In addition, fecundity assays showed that a similar number of phage particles were present in the plaques generated by each of the mutants and by the wild-type phage (data not shown). Taken together, these results suggest that none of these genes is required for lytic growth of TM4 or for efficient phage propagation.

### Mycobacteriophage genes associated with mycobacterial growth inhibition are expressed during lytic growth of mycobacteriophage TM4.

**FIG 5:**
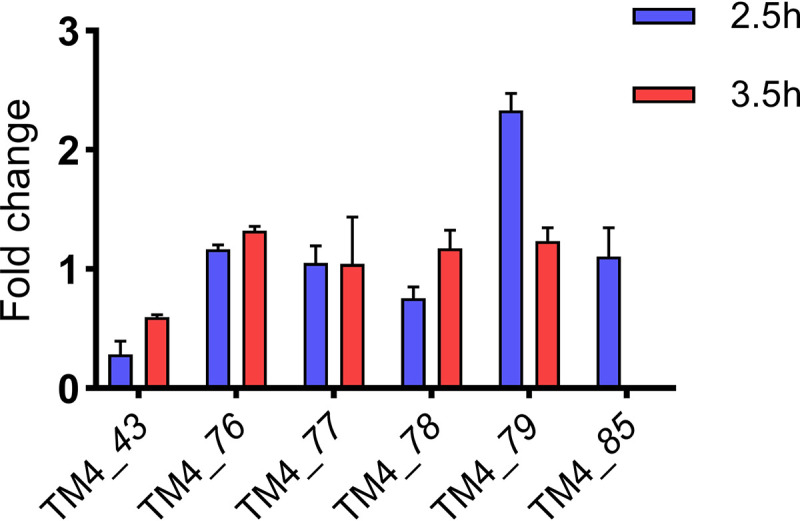
Transcription levels of TM4 genes at different infection phases. RNA was isolated from cultures of M. smegmatis infected with TM4 at an MOI of 3, at 1, 2.5, and 3.5 h after infection, and the expression level for each of the genes indicated was determined by qRT-PCR. The fold changes of mRNA levels for each gene at 2.5 and 3.5 h relative to mRNA levels at 1 h after infection are shown. The results represent the means ± standard deviations of three replicates and are representative of at least two independent experiments.

Although the mycobacteriophage genes associated with mycobacterial growth inhibition are not required for lytic growth, they may be involved in influencing phage-host dynamics, including exclusion. Thus, the pattern of expression of these genes was analyzed following infection of M. smegmatis. The expression of gene *79* at 2.5 h postinfection was significantly higher than at 1 h postinfection, whereas the expression of gene *43* at 2.5 h and 3.5 h postinfection was significantly lower than at 1 h postinfection (Fig. 5). The expression of the remaining genes did not significantly change during lytic growth of mycobacteriophage TM4 (Fig. 5).

## DISCUSSION

Identification and characterization of phage-carried genes encoding proteins toxic to mycobacteria could lead to the identification of drug targets for novel antibiotic development. To date, >10,000 mycobacteriophages have been isolated. The significant genetic diversity of these phages provides a rich source of potential targets but also poses challenges for the screening of the genes of interest (http://phagesdb.org/). In this study, we described a high-throughput method, based on NGS, for the screening of mycobacteriophage genes associated with inhibition of mycobacterial growth, and we applied this method to mycobacteriophage TM4. This revealed three TM4 regions, including five genes, *43*, *77*, *78*, and *79*, and *85*, which inhibited the growth of M. smegmatis when overexpressed. Gene *43* is a homologue of the gp34 gene in mycobacteriophage D29, which encodes an excise protein suggested to be required for phage DNA excision from the bacterial chromosome (21). Since expression of gp43 was not toxic to E. coli or M. tuberculosis, this protein might recognize and digest DNA sequences specific to M. smegmatis and other mycobacteria. The genes *77*, *78* and *79* encode three, small, nonessential proteins with no predicted functions (24), and they are only toxic to mycobacteria when they were coexpressed. In addition, expression of these genes is also nontoxic in E. coli, indicating that the proteins might be involved in mycobacterium-specific pathways, which have yet to be elucidated. gp85, a 156-amino-acid protein with no conserved domains and no known function, exhibited an antibacterial effect in E. coli, M. smegmatis, and M. tuberculosis.

A genome library representing several mycobacteriophages was constructed previously to screen for genes associated with antimycobacterial activity (21). In that study, phage DNA fragments of 500 to 700 bp or 700 to 1,500 bp were cloned into a plasmid containing an inducible promoter and expressed in M. smegmatis. Screening of the growth of about 3,000 transformed colonies of M. smegmatis, under induced and uninduced conditions, identified two genes from mycobacteriophage D29 and Che12 whose expression inhibited M. smegmatis, but that study failed to find any such genes in either TM4 phage or 3 other mycobacteriophages. Two reasons might account for why this study did not identify any inhibitory genes in TM4: (i) the cloned genes may have been expressed from their own promoters, making them toxic to M. smegmatis even under uninduced conditions; or (ii) the constructed library did not have sufficient coverage of the phage genomes, and achieving high coverage would be very labor-intensive using their method. In our study, NGS analysis was used to give 1,000× coverage of the TM4 library, thus reducing the amount of labor required to identify genes toxic to E. coli and M. smegmatis. Because our method can rapidly narrow the screening to genes associated with bacterial toxicity, it may be suitable for large-scale screening of toxic genes not only in mycobacteriophages but also in other phages.

The production of toxic proteins might disrupt the normal metabolic pathways required for phage replication; thus, the expression of toxic proteins in lytic growth is unlikely to be directly beneficial to the phage (27–32). Consistent with this hypothesis, none of the TM4 genes associated with antimycobacterial activity was required for lytic growth and propagation of phage. The phage genes associated with bacterial toxicity might play important roles in phage competition in their natural environments. For example, gp52 of mycobacteriophage Fruitloop interacts directly with DivIVA, an essential host protein, and might in turn interfere with superinfection by a DivIVA-dependent phage (33). Nevertheless, the antibacterial or bactericidal mechanisms of toxic mycobacteriophage proteins and their physiological roles are still poorly understood. Thus, the identification of novel, phage-derived, toxic proteins and the elucidation of their roles will provide key insights into host-phage dynamics. Our NGS-based high-throughput screening method will facilitate identification of novel genes encoding such proteins and should shed light on the interactions between mycobacteriophages and their hosts. This, in turn, could contribute to the development and improvement of antituberculosis drugs.

## MATERIALS AND METHODS

### Strains, phage, plasmids, media, and growth conditions.

E. coli was grown in Luria-Bertani (LB) medium supplemented with appropriate antibiotics. M. smegmatis mc^2^155 was grown at 37°C in Middlebrook 7H9 broth (BD Biosciences) supplemented with 0.2% (wt/vol) glycerol (Sigma) and 0.05% (wt/vol) Tween 80 (Sigma) or on Middlebrook 7H10 agar (BD Biosciences) supplemented with 0.5% (wt/vol) glycerol. M. tuberculosis H37Ra was grown in Middlebrook 7H9 medium supplemented with 10% oleic acid-albumin-dextrose-catalase (OADC) and 0.05% Tween 80, or on Middlebrook 7H10 agar supplemented with 10% OADC. If antibiotic selection was necessary, 25 μg/mL kanamycin, 50 μg/mL zeocin, or 50 μg/mL hygromycin was added to Middlebrook 7H9 or Middlebrook 7H10, and 50 μg/mL kanamycin, 25 μg/mL zeocin, or 100 μg/mL hygromycin was added into LB broth medium. Anhydrotetracycline (ATc) was used at a concentration of 100 ng/mL, unless otherwise specified. When cultures were used for phage infection, Tween 80 was replaced with 1 mM CaCl_2_.

The expression of TM4 genes in E. coli and M. smegmatis mc^2^155 was achieved by cloning TM4 DNA fragments into the vector pBAD/Myc-HisA or pYC601 (34), respectively, using a homologous recombination seamless cloning kit (catalogue number CU101-02, TransGen Biotech).

Phasmid phAE87 was used as the wild type of mycobacteriophage TM4 (25). Mutants of TM4 were constructed using the CRISPR/Cas12a-based plasmid editing method described previously (35). Briefly, phasmid phAE87, crRNA-expressing plasmid (Table S2), and oligonucleotides (Table S3) were electroporated into SY4539 competent cells in which Cas12a and λ Red recombination proteins had been induced. Phasmid mutants were verified by sequencing.

The strains, phages, plasmids, and primers used in this study are listed in Tables S2 and S3 in the supplemental material.

### Construction of the mycobacteriophage TM4 DNA library.

The mycobacteriophage TM4 genome was partially digested with 0.5 U Sau3AI (catalogue number R0169S, NEB) for 10 min at 37°C, and the reaction was terminated by heat inactivation (65°C, 20 min). DNA fragments in the 3-to-10-kb size range were gel purified and ligated into a pMV261 vector, which had been digested with BamHI and dephosphorylated with calf intestinal alkaline phosphatase (NEB). The ligation products were transformed into E. coli Trans T1, and more than 10,000 transformants were obtained. Eight transformants were picked for PCR analysis, and six of them were shown to harbor insertion fragments larger than 3 kb. Recombinant plasmid mixtures were extracted from E. coli and then electroporated into M. smegmatis mc^2^155, resulting in more than 10,000 transformants.

### Next-generation sequencing and data analysis.

Recombinant plasmids mixtures were purified from E. coli and M. smegmatis and then subjected to NGS analysis at Azenta Life Sciences. Briefly, purified plasmids were fragmented and then treated to repair the fragmented ends and add a d(A) tail in one reaction, followed by a T-A ligation to add adaptors to both ends. Size selection of adaptor-ligated DNA was then performed using DNA Clean beads. Each sample was then amplified by PCR using P5 and P7 primers, and the PCR products were validated. Then, libraries with different indices were multiplexed and loaded on an Illumina HiSeq/Illumina Novaseq/MGI2000 instrument for sequencing using a 2-by-150 paired-end configuration according to the manufacturer’s instructions. To analyze the sequencing data, reference genome sequences were obtained from the NCBI genome website (NC_003387.1). The sequencing data were aligned to reference genome via software Hisat2 (v2.0.1) and HTSeq (v0.6.1). Differential expression was analyzed using the DESeq2 Bioconductor package.

### Lytic growth and fecundity assay.

For the lytic growth assay, ~200 ng DNA of TM4 phasmid derivatives was electroporated into M. smegmatis mc^2^155, and phage plaque formation was observed by the bilayer agar method as described previously (33). For the fecundity assay, three phage plaques from the lytic growth assay were randomly chosen. The number of phage particles contained in one of the phage plaques was analyzed as described previously (33).

### Growth curve assay.

For growth assays of M. smegmatis, M. smegmatis mc^2^155 cells harboring the pYC601 vector, with or without cloned TM4 inserts, were grown in 7H9 culture medium at 37°C at 200 rpm until stationary phase was reached, and then aliquots were transferred into fresh 7H9 medium supplemented with or without 100 ng/mL ATc to give an initial optical density at 600 nm (OD_600_) of ~0.01. Cultures were further incubated at 37°C with shaking, and OD_600_ values were measured every 4 h. For growth assays of E. coli, E. coli cells harboring the pBAD vector, with or without cloned TM4 inserts, were grown in LB medium at 37°C until stationary phase was reached. Aliquots were transferred to fresh LB medium supplemented with or without 0.1% (wt/vol) arabinose to give an initial OD_600_ of ~0.01. Cultures were further incubated at 37°C with shaking, and OD_600_ values were measured every 0.5 h. The experiments were performed three times independently, each time with two biological replicates.

### RNA isolation and qRT-PCR.

M. smegmatis cells were cultured to an OD_600_ of ~0.7 and infected with TM4 phage at a multiplicity of infection of 3. Cells were harvested at 1 h, 2 h, and 3.5 h postinfection, and RNA was extracted using the RNeasy minikit (catalogue number 74106, Qiagen) according to the manufacturer's instructions. Reverse transcription-quantitative PCR (qRT-PCR) was performed using *Taq* Pro Universal SYBR qPCR master mix (catalogue number Q712, Vazyme). Data were normalized for 16S RNA gene expression levels. The threshold cycle (2^−ΔΔ^*^CT^*) method was used to analyze relative changes in gene expression.

### Data availability.

The data that support these findings are available from the corresponding author upon reasonable request. Raw sequencing reads from NGS in this study have been deposited at the NCBI Sequence Read Archive with the accession code PRJNA907983.
