# Supplementary material for: Identification of Toxic Proteins Encoded by Mycobacteriophage TM4 Using a Next-Generation Sequencing-Based Method
Source: Microbiol Spectr. 2023 May 8;11(3):e05015-22. doi: 10.1128/spectrum.05015-22 (PMC10269906; doi:10.1128/spectrum.05015-22)
Supplement: Supplemental file 1 — Tables S1 to S3 and Fig. S1 to S3. Download spectrum.05015-22-s0001.pdf, PDF file, 0.4 MB [file spectrum.05015-22-s0001.pdf]

Supplementary Materials

Identification of toxic proteins encoded by mycobacterophage TM4 using a next generation sequencing-based method

Chun-Liang Wang[a], Lan-Yue Zhang[b], Xin-Yuan Ding[a], Yi-Cheng Sun[a#]

#Corresponding author. sunyc@ipbcams.ac.cn

Table S1. The gene-specific reads percentages of plasmids mixtures from *E. coli* and plasmid mixtures from *M. smegmatis*, and their corresponding ratios.

Gene	% Reads in plasmids mix from *M. smegmatis*	% Reads in plasmids mix from *E. coli*	Log2 (Ratio)
TM4_gp1	0.0381	0.0284	0.4256
TM4_gp2	0.0060	0.0054	0.1541
TM4_gp3	0.0201	0.0106	0.9256
TM4_gp4	4.4777	3.1282	0.5174
TM4_gp5	4.5482	3.4341	0.4054
TM4_gp6	5.5998	4.7656	0.2327
TM4_gp7	0.0471	0.0457	0.0435
TM4_gp8	1.2789	1.2152	0.0737
TM4_gp9	3.0452	2.8431	0.0991
TM4_gp10	0.5780	0.5444	0.0862
TM4_gp11	0.2219	0.1829	0.2789
TM4_gp12	0.0351	0.0270	0.3776
TM4_gp13	0.7920	0.7897	0.0041
TM4_gp14	1.9837	1.8925	0.0679
TM4_gp15	1.1832	0.9575	0.3052
TM4_gp16	0.8982	0.5887	0.6095
TM4_gp17	17.4070	12.2426	0.5078
TM4_gp18	4.9931	3.4846	0.5190
TM4_gp19	7.4837	5.4784	0.4500
TM4_gp20	0.9901	0.8542	0.2130
TM4_gp21	3.2856	2.9474	0.1567
TM4_gp22	0.0281	0.0254	0.1424
TM4_gp23	10.5111	8.5441	0.2989
TM4_gp24	0.2027	0.1618	0.3248
TM4_gp25	4.0688	3.5032	0.2159
TM4_gp27	1.3922	1.4408	-0.0495
TM4_gp29	4.9518	5.1748	-0.0636
TM4_gp30	3.0923	3.4728	-0.1674
TM4_gp31	0.2663	0.3104	-0.2212
TM4_gp33	0.3408	0.3735	-0.1320
TM4_gp34	0.4914	0.6505	-0.4045
TM4_gp35	0.0141	0.0281	-0.9920
TM4_gp36	1.6587	3.2753	-0.9816
TM4_gp37	0.0015	0.0047	-1.6394

TM4_gp38	0.1902	0.3544	-0.8978
TM4_gp39	0.1774	0.3375	-0.9282
TM4_gp40	0.9359	2.7912	-1.5764
TM4_gp41	0.0758	1.8057	-4.5735
TM4_gp42	0.0146	1.3095	-6.4901
TM4_gp43	0.0075	0.0242	-1.6885
TM4_gp44	0.0075	0.0728	-3.2789
TM4_gp45	0.0062	0.0480	-2.9491
TM4_gp46	0.0176	0.0733	-2.0612
TM4_gp47	0.0109	0.0251	-1.1981
TM4_gp48	0.6855	1.4971	-1.1270
TM4_gp49	0.0122	0.0221	-0.8560
TM4_gp50	0.8363	1.3066	-0.6437
TM4_gp51	0.0002	0.0017	-2.9943
TM4_gp52	0.8132	1.1232	-0.4660
TM4_gp53	0.1943	0.2753	-0.5026
TM4_gp54	0.1510	0.2047	-0.4384
TM4_gp56	0.0047	0.0050	-0.0943
TM4_gp57	1.5816	1.9368	-0.2922
TM4_gp59	2.0291	2.4430	-0.2678
TM4_gp60	0.0066	0.0040	0.7160
TM4_gp61	0.0985	0.0993	-0.0107
TM4_gp62	0.2757	0.3198	-0.2142
TM4_gp64	0.0184	0.0341	-0.8900
TM4_gp65	0.0092	0.0124	-0.4286
TM4_gp66	0.5090	0.9448	-0.8923
TM4_gp67	0.0388	0.0725	-0.9029
TM4_gp68	0.0045	0.0023	0.9455
TM4_gp69	0.0985	0.1986	-1.0114
TM4_gp70	3.7442	6.2580	-0.7410
TM4_gp71	0.0229	0.0378	-0.7226
TM4_gp72	0.3616	0.6970	-0.9467
TM4_gp73	0.0030	0.0029	0.0610
TM4_gp74	0.0315	0.0624	-0.9876
TM4_gp75	0.1680	0.9862	-2.5538
TM4_gp76	0.0041	0.2212	-5.7640
TM4_gp77	0.0001	0.0172	/
TM4_gp78	0.0009	0.0208	-4.5981
TM4_gp79	0.0011	0.0717	-6.0647
TM4_gp80	0.0443	0.1388	-1.6463
TM4_gp81	0.0351	0.0528	-0.5885

TM4_gp82	0.1365	0.3216	-1.2370
TM4_gp83	0.0069	0.0092	-0.4188
TM4_gp84	0.0004	0.0459	-6.7435
TM4_gp85	0.0017	0.3660	-7.7386
TM4_gp86	0.0422	0.3080	-2.8675
TM4_gp87	0.1864	0.2259	-0.2773
TM4_gp88	0.0002	0.0004	-1.0683
TM4_gp89	0.0092	0.0149	-0.6951
TM4_gp90	0.0947	0.0737	0.3621
TM4_gp91	0.0056	0.0033	0.7446
TM4_gp92	0.3556	0.2875	0.3068

Table S2. **The strains, phages, plasmids used in this study**

Name	Description	References
**Bacterial strains**		
E. coli Trans1-T1	Expression host	TransGen Biotech
SY4539	E. coli strain carries Cas12a expression cassette for plasmid editing	[1, 2]
M. smegmatis MC^{2}155	Wild-type	[3]
M. Tuberculosis H37Ra	Wild-type	Lab stock
**Phages**		
phAE87	Mycobacteriophage TM4 phasmid	[4]
TM4_mut*43*	Gene *43* was mutated by introduction of a stop codon	This study
TM4_mut*76*	The gene *76* of TM4 was mutated by deletion of 50 bp	This study
TM4_mut*77*	The gene *77* of TM4 was mutated by deletion of 50 bp	This study
TM4_mut*78*	The gene *78* of TM4 was mutated by deletion of 50 bp	This study
TM4_mut*79*	The gene *79* of TM4 was mutated by deletion of 50 bp	This study
TM4_mut*85*	The gene *85* of TM4 was deleted for 50 bp	This study
**Plasmids**		
pBAD/Myc-HisA	*E. coli* expression plasmid with an arabinose-inducible promoter	Invitrogen
pYC601	Plasmid with tetracycline inducible promoter for expression in mycobacteria	[5]
pMV261	Shuttle vector, replicate extrachromosomal in *E. coli* and mycobacterium	[6]
pAC-crRNA	For plasmid editing, crRNA-expressing plasmid	[1]

Table S3. **The primers and oligonucleotides used in this study**

Primer or oligonucleotide name	Sequence	Description
gp41-pYC601-F	TCCGCATGCGGAGGAATCAGGATCCAGTGCGCCGGTGTCCACACGC	For clone TM4_*41* into pYC601 forward primer
gp41-pYC601-R	GTCCCCAATTAATTAGCTAAAGCTTTCAGGCGCGCGTCGCGTT	For clone TM4_*41* into pYC601 reverse primer
gp42-pYC601-F	TCCGCATGCGGAGGAATCAGGATCCAATGGTGCAGATTCTGGACAAGTG	For clone TM4_*42* into pYC601 forward primer
gp42-pYC601-R	GTCCCCAATTAATTAGCTAAAGCTTTCATAGCAGCACCGTCGCGC	For clone TM4_*42* into pYC601 reverse primer
gp43-pYC601-F	TCCGCATGCGGAGGAATCAGGATCCAATGACCGGCGCCTTGCTGA	For clone TM4_*43* into pYC601 forward primer
gp43-pYC601-R	GTCCCCAATTAATTAGCTAAAGCTTTCAGCCCCTAATCCGGCGTT	For clone TM4_*43* into pYC601 reverse primer
gp76-pYC601-F	TCCGCATGCGGAGGAATCAGGATCCAATGACCGAAATCACAGCAGGTT	For clone TM4_*76* into pYC601 forward primer
gp76-pYC601-R	GTCCCCAATTAATTAGCTAAAGCTTTCACTTCTCGATCCGATCGTG	For clone TM4_*76* into pYC601 reverse primer
gp77-78-79-pYC601-F	TCCGCATGCGGAGGAATCAGGATCCAGTGAAGCGCACCAGGACGGT	For clone TM4_*77-79* into pYC601 forward primer
gp77-78-79-pYC601-R	GTCCCCAATTAATTAGCTAAAGCTTCTAGTCGCGGGCCATGTGG	For clone TM4_*77-79* into pYC601 reverse primer
gp84-pYC601-F	TCCGCATGCGGAGGAATCAGGATCCAGTGCTGAGCGTGCAGCCC	For clone TM4_*84* into pYC601 forward primer
gp84-pYC601-R	GTCCCCAATTAATTAGCTAAAGCTTCTACGGGTCGAACTCCTCG	For clone TM4_*84* into pYC601 reverse primer
gp85-pYC601-F	TCCGCATGCGGAGGAATCAGGATCCAATGTACAAGATGATCGTTCAAATGT	For clone TM4_*85* into pYC601 forward primer
gp85-pYC601-R	GTCCCCAATTAATTAGCTAAAGCTTTCAGGCGGCCAGCGTCAG	For clone TM4_*85* into pYC601 reverse primer
gp43-pBAD-F	TTTGGGCTAACAGGAGGAATTAACCATGACCGGCGCCTTGCTGA	For clone TM4_*43* into pBAD forward primer
gp43-pBAD-R	TGAGTTTTTGTTCGGGCCCAAGCTTTCAGCCCCTAATCCGGCGTT	For clone TM4_*43* into pBAD reverse primer
gp76-pBAD-F	TTTGGGCTAACAGGAGGAATTAACCATGACCGAAATCACAGCAGGTT	For clone TM4_*76* into pBAD forward primer
gp76-pBAD-R	TGAGTTTTTGTTCGGGCCCAAGCTTTCACTTCTCGATC	For clone TM4_*76* into pBAD reverse

	CGATCGTG	primer
gp77-78-79-pBAD-F	TTTGGGCTAACAGGAGGAATTAACCGTGAAGCGCACCAGGACGGT	For clone TM4_*77-79* into pBAD forward primer
gp77-78-79-pBAD-R	TGAGTTTTTGTTCGGGCCCAAGCTTCTAGTCGCGGGCCATGTGG	For clone TM4_*77-79* into pBAD reverse primer
gp84-pBAD-F	TTTGGGCTAACAGGAGGAATTAACCGTGCTGAGCGTGCAGCCC	For clone TM4_*84* into pBAD forward primer
gp84-pBAD-R	TGAGTTTTTGTTCGGGCCCAAGCTTCTACGGGTCGAACTCCTCG	For clone TM4_*84* into pBAD reverse primer
gp85-pBAD-F	TTTGGGCTAACAGGAGGAATTAACCATGTACAAGATGATCGTTCAAATGT	For clone TM4_*85* into pBAD forward primer
gp85-pBAD-R	TGAGTTTTTGTTCGGGCCCAAGCTTTCAGGCGGCCAGCGTCAG	For clone TM4_*85* into pBAD reverse primer
gp43-oligo	cagacttgcgacccgactcgggggcgactcagaacgattcgagagcggcctctatgtcg	For gene *43* mutation
gp76-oligo	cggcgtagccgatcagcgtcacgacgtcggccttactggcgcaaacctgctgtgatttcggtcatttcgtgggctcct	For gene *76* mutation
gp77-oligo	acgcctcatgcccggccgggccgccaatgaagtcgagcacgctcgccgcggatcgacacctcggtgcccggctcaagc	For gene *77* mutation
gp78-oligo	cgcgtttcgtggtggtcagtcgtcggtcagctcgggcagcggcggccacctgagccccgagggcgaccaccagggcca	For gene *78* mutation
gp79-oligo	cgacacgcgagtgcggccgacgagcacgacctcgccgtaggccgacggcgaactcaacagccgactcgccgtaaacgt	For gene *79* mutation
gp85-oligo	cgtaacggcgatcgtgaccaggcgctcgcgggcctcggctcttgtacattttgggctcctatcccgtcgtgctgttga	For gene *85* mutation

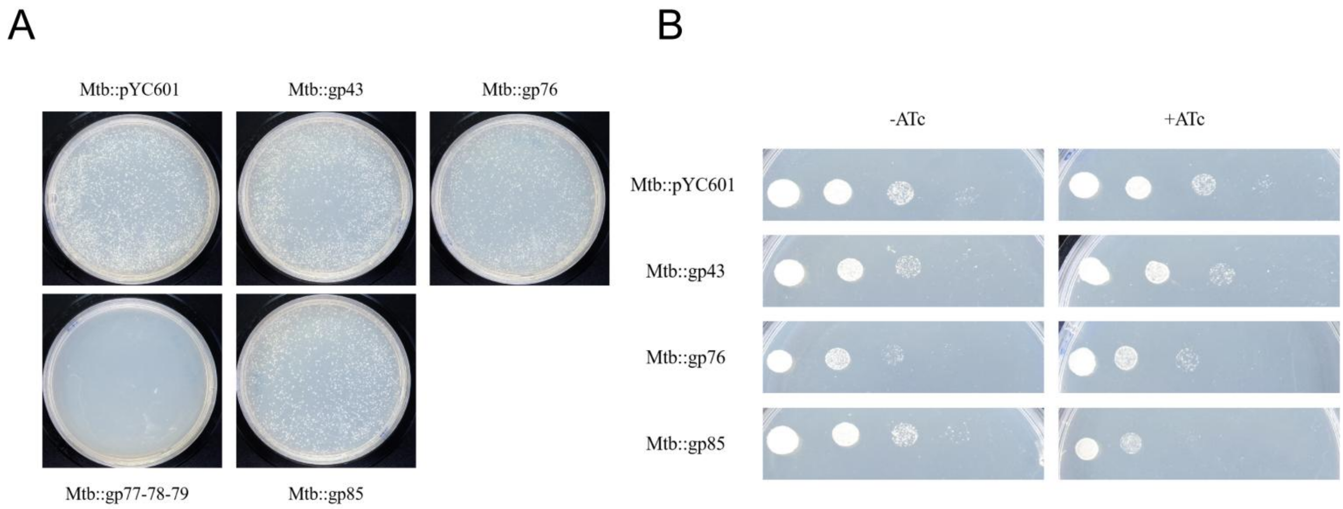

Fig S1 Expression of genes *77-78-79* or *85* inhibited the growth of *M. tuberculosis.* (A) Expression plasmids of the corresponding phage genes were electrotransduced to *M. tuberculosis*, resuscitated for 24 h and coated with 7H10 plates (50 μg/mL hygromycin were added). (B) *M. tuberculosis* transformants carrying an empty expression vector (pYC601) or pYC601 containing the cloned mycobacteriophage genes specified were gradient diluted and spotted on 7H10 agar with (+ATc) or without (−ATc) inducer. ATc was added at a concentration of 100 ng/ml.

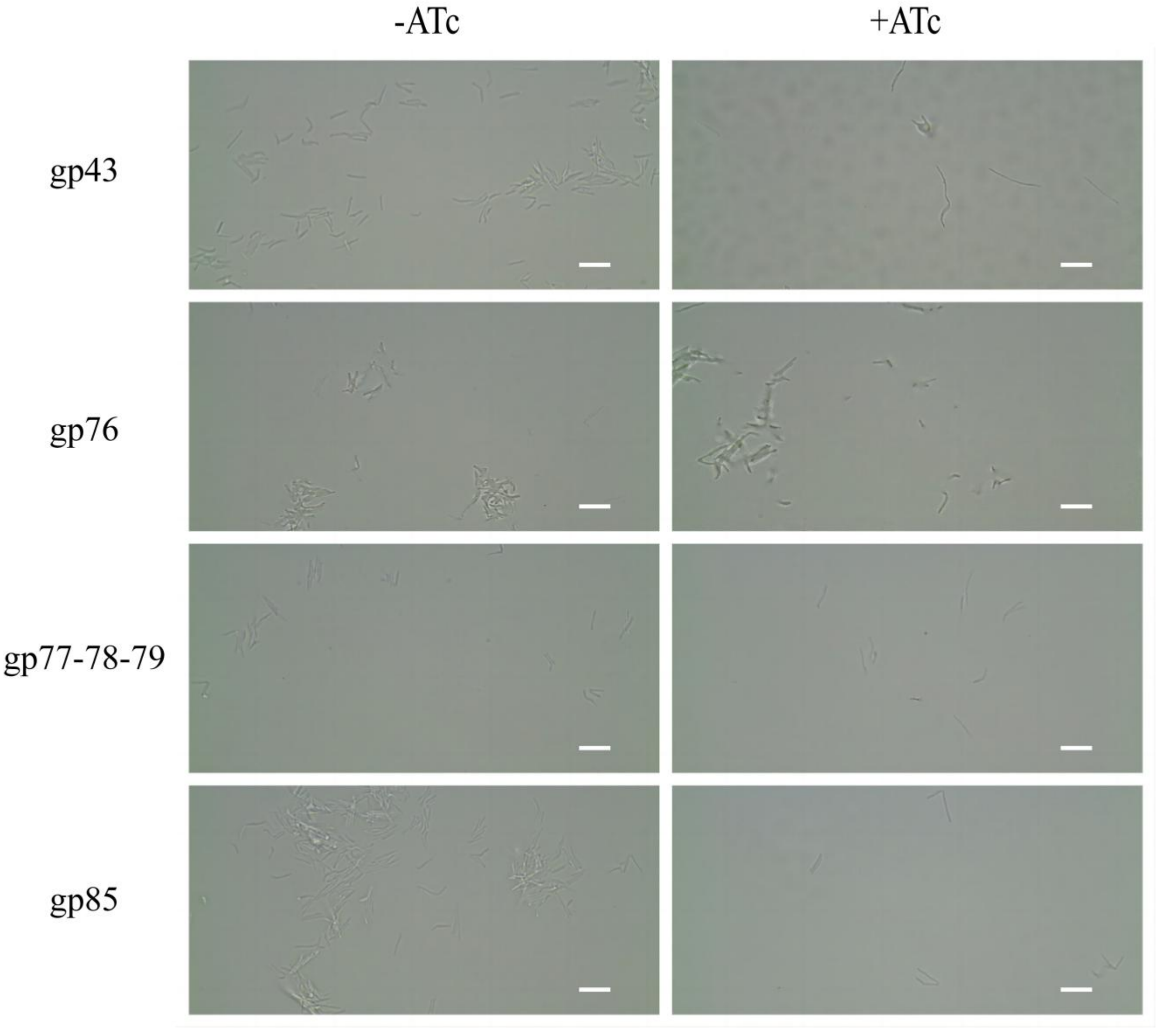

Fig S2 Cellular morphologies following induction of toxic gene expression. *M. smegmatis* transformants carrying mycobacteriophage toxic genes were examined by bright field microscopy 12 h after induction. The scale bar corresponds to 5 μm.

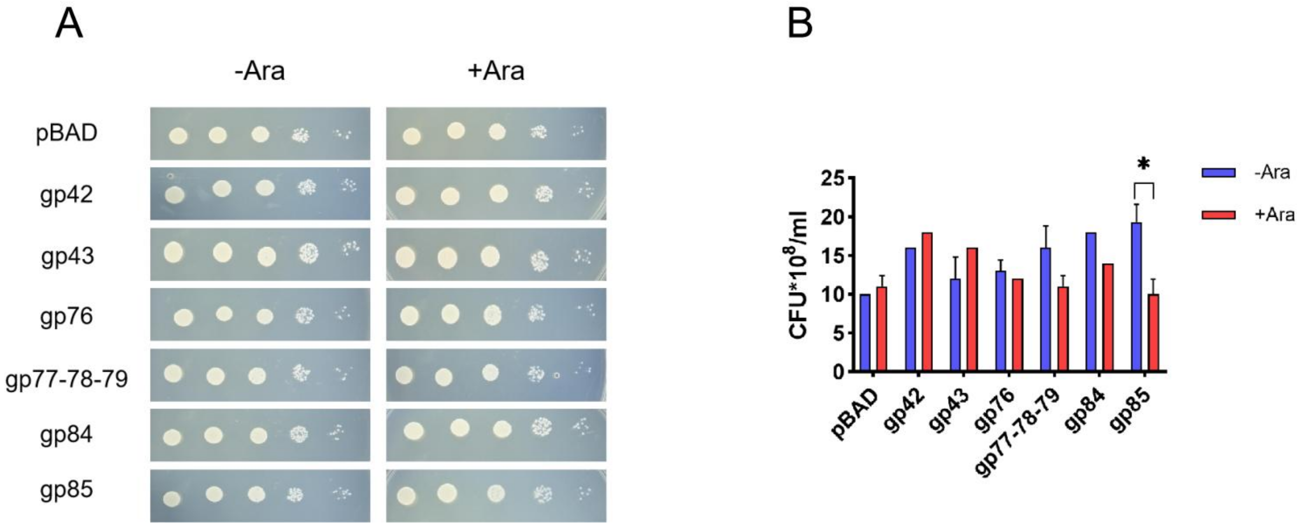

Fig S3 Expression of gene *85* of mycobacteriophage TM4 inhibits growth of *E. coli.*(A) *E. coli* transformants carrying an empty expression vector (pBAD) or pBAD containing the cloned mycobacteriophage genes specified were gradient diluted and spotted on LB agar with (+Ara) or without (−Ara) inducer. Arabinose was added at a concentration of 0.1%. (B) Quantitative analysis of the dot-plate experiments. The experiments were repeated three with similar results and one experiment result is shown. * $P < 0.05$.

References:

[1]. Geng, Y., et al., A highly efficient in vivo plasmid editing tool based on CRISPR-Cas12a and phage λ Red recombineering. Journal of Genetics and Genomics, 2019. 46(9): p. 455-458.

[2]. Sawitzke, J.A., et al., Recombineering: highly efficient in vivo genetic engineering using single-strand oligos. Methods Enzymol, 2013. 533: p. 157-77.

[3]. Snapper, S.B., et al., Isolation and characterization of efficient plasmid transformation mutants of Mycobacterium smegmatis. Mol Microbiol, 1990. 4(11): p. 1911-9.

[4]. Bardarov, S., et al., Conditionally replicating mycobacteriophages: a system for transposon delivery to Mycobacterium tuberculosis. Proc Natl Acad Sci U S A, 1997. 94(20): p. 10961-6.

[5]. Mao, X.J., et al., Efficient and simple generation of multiple unmarked gene deletions in Mycobacterium smegmatis. Sci Rep, 2016. 6: p. 22922.

[6]. Stover, C.K., et al., New use of BCG for recombinant vaccines. Nature, 1991. 351(6326): p. 456-60.
